# Multi-cellularity in cardiac tissue engineering, how close are we to native heart tissue?

**DOI:** 10.1007/s10974-019-09528-8

**Published:** 2019-06-20

**Authors:** Thomas J. Owen, Sian E. Harding

**Affiliations:** National Heart and Lung Institute, Imperial College London Hammersmith Campus, Imperial Centre for Translational and Experimental Medicine, Du Cane Road, London, W12 0NN UK

**Keywords:** Tissue engineering, Cardiomyocyte, Stem cell, Contraction, Multi-cellularity

## Abstract

Tissue engineering is a complex field where the elements of biology and engineering are combined in an attempt to recapitulate the native environment of the body. Tissue engineering has shown one thing categorically; that the human body is extremely complex and it is truly a difficult task to generate this in the lab. There have been varied attempts at trying to generate a model for the heart with numerous cell types and different scaffolds or materials. The common underlying theme in these approaches is to combine together matrix material and different cell types to make something similar to heart tissue. Multi-cellularity is an essential aspect of the heart and therefore critical to any approach which would try to mimic such a complex tissue. The heart is made up of many cell types that combine to form complex structures like: deformable chambers, a tri-layered heart muscle, and vessels. Thus, in this review we will summarise how tissue engineering has progressed in modelling the heart and what gaps still exist in this dynamic field.

The myocardium is a complex tissue consisting of many different cell types working together. The tissue engineering field has attempted to model this in the lab with varying levels of success. Early approaches in the cardiac field took the lead from the muscle field and produced contracting 3D matrixes (Eschenhagen and Zimmermann 2005). Much of this body of work used isolated neonatal or embryonic cardiomyocytes which were then joined together by a scaffold. These seminal pieces of work were quite successful, but the question remained about which kind of cells would be used going forward. More recently the field has shifted to pluripotent stem cells (PSCs) which hold much promise for tissue engineering because they can be differentiated into multiple cell types and they are a potentially unlimited supply of cells. Moreover, adult cells cannot be cultured in vitro for longer than a few days so PSCs represent a more amenable model for the lab. However, there is some discussion about which cells would be most appropriate for generation of tissue constructs in the lab. Induced pluripotent stem cells can be derived from adult patients which makes them less ethically questionable compared with embryonic stem cells, and a potential source for autologous transplantation or patient specific disease modelling. Although, there has been some discussion in the field about how similar these cell types are to each other, and which source may be considered best for applications in the lab (Bilic and Izpsua Belmonte 2012).

Tissue engineering has advanced and the combination of biology and engineering have produced different materials capable of fabricating scaffolds. These engineered materials can be manipulated and researchers can now adjust many properties of: collagen, alginate, gelatin sponges, polyhydroxyalkanoate, polyvinyl acetate, and polyglycolic acid derivatives (Radisic et al. 2004; Leor et al. 2000; Li et al. 1999; Carrier et al. 1999; Lesman et al. 2010; Marsano et al. 2010; Tang et al. 2018; Basnett et al. 2018). These scaffolds can be made in large enough quantities to carry out studies using pluripotent stem cell derived cardiomyocytes (PSC-CM) and some improvements to cardiomyocyte phenotype have been reported. Moreover, additional stimulation can also be applied to make the environment more similar to the heart including electrical stimulation, stretch and different hormonal signals. Constructs made using these combined approaches are then assessed for their similarity to adult cardiomyocytes or heart tissue. There are many factors that are assessed for hPSC-CM maturation in the areas of structure, electrical activity, force generation and pharmacological response, with some reviews having as many as 32 parameters (Denning et al. 2016). Pseudo tissues are tested for their similarity to heart tissue with these factors in mind and specific parameters which involve many cells like force generation, and conduction from one point to another. Although improvements and similarities have been reported the field is still a long way from making true myocardium. The addition of cell types other than cardiomyocytes is an important strategy to improve the fidelity of tissue engineered myocardium. Currently there are many under investigation.

## Fibroblasts critical modulators of the heart

Cardiac fibroblasts are a critical component of the heart and as such have been intensively studied in tissue engineering approaches. Fibroblasts in the heart lay down extracellular matrix and continue to regulate this dynamic structure, in addition to this they are also involved with mechanical support, electrical conduction, and paracrine signalling (Abramochkin et al. 2014; Cartledge et al. 2015; Shinde and Frangogiannis 2014). In the native heart the contribution of fibroblasts to total cell number is around 20–50% (Jugdutt 2003; Porter and Turner 2009; Krenning et al. 2010; Pinto et al. 2016), with recent reports now indicating endothelial cells are the most abundant non-cardiomyocyte cell type (Pinto et al. 2016).

## Tissue engineering with fibroblasts

Tissue engineering approaches have shown that fibroblasts make engineered tissues more similar to myocardium. The addition of fibroblasts at 10–30% has been shown to make constructs thinner bringing cardiomyocytes closer together (Liau et al. 2011; Kenash et al. 2013; Liau et al. 2017; Tiburcy et al. 2017). This has the effect on increasing force produced by these constructs. Tiburcy and colleagues also showed that there is an optimal amount of fibroblasts that can be added which relies on fibroblasts proliferating during the maturation phase of engineering tissues. Moreover, when constructs have reached peak force production the cell contribution of fibroblasts to cardiomyocytes is at a roughly 1:1 ratio which is similar to some measurements of cell contributions in adult hearts (Tiburcy et al. 2017). The use of fibroblasts aided the maturation of cells in their morphology, twitch forces, positive force frequency responses and gene regulation. Furthermore, the type of the fibroblast being used is also important with some studies indicating that fibroblast identity is lost within a few passages (Rohr 2011; Cartledge et al. 2015). Both adult and foetal fibroblasts have been investigated for their effect on engineered constructs and showed that foetal cells are better than adult cells based on multiple parameters (Li et al. 2017). These results show that fibroblasts are key to engineered heart constructs and these experiments were the first to demonstrate that additional cell types can improve cardiac tissues by having a direct effect on iPSC-CM.

## Cells involved in vessel formation

The ratio of three endothelial cells to one cardiomyocyte is usually cited to show the significance of this cell type within the heart. The heart is a mechanically active organ with a high demand for oxygen, therefore it needs an effective blood supply. This is particularly important in tissue engineering due to the limits of oxygen diffusion: an in vitro model cannot be thicker than a few 100 μm, otherwise a necrotic core will develop. Endothelial cells are the basic building block of vessels with interactions between the extra cellular matrix, smooth muscle cells and pericytes being critical to this process (Sweeney and Foldes 2018). Tube structures can be formed in vitro but mature vessels which can handle similar pressures to in vivo have proved difficult to obtain. This also adds a layer of complexity to the engineering problem because a functional myocardium must be made with an adequate vasculature.

## Tissue engineering using endothelial cells

There have been many approaches which use tissue engineering to create an in vitro angiogenesis model, and these have produced a great deal of information about how these cells respond to different environments. In vitro models have used matrices such as fibrin, collagen or Matrigel with similar levels of success (Sun et al. 2016). Neonatal rat cardiomyocytes loaded in Matrigel and placed in vivo can be vascularised and mature, which demonstrates that immature cells given the correct cues can form tissue with similar properties to myocardium (Morritt et al. 2007). Cells directly isolated from mice and then cultured in fibrin hydrogels show that relatively large vessels can be formed within a hydrogel (Stoehr et al. 2016). In addition, cells can migrate in-between veins and arteries and form mature capillaries within a hydrogel which acted as a pre-made vascular bed for a cardiomyocyte hydrogel to be formed around (Chiu et al. 2012). Importantly, this vascular bed was perfusable, which demonstrates a necessary feature in vasculature, and is lacking in many approaches in this field.

Tissue engineering approaches have an important consideration for regenerative medicine since ischaemic cardiomyopathy and myocardial infarction are caused by lack of blood flow to the ventricles. In addition, it is important to consider vessel formation when considering using engineered patches for treatments, therefore, when regarding regenerative medicine the argument for inclusion of cells to produce vessels is clear. However, there is some discussion whether endothelial cells are needed for the patch approach. Comparisons of dual or tri-cultured tissue constructs which have been engrafted onto animal models with endothelial cells show increased CD31^+^ vessel structures inside the hydrogel (Lesman et al. 2010; Tulloch et al. 2011). This could be very important for heart disease especially since vascularised grafts are more likely to survive long term, and increasing blood flow to the infarcted area could help recovery substantially. Recently, a paper from Thomas Eschenhagen’s group tested adding endothelial cells into their fibrin engineered tissues in a guinea-pig model of myocardial infarction (Weinburger et al. 2016). The endothelial cell constructs had no effect on left ventricular function and performed similarly to cell free constructs. Therefore, these papers show that a fine balance must be found between cardiomyocytes and non-cardiomyocytes in patches for regenerative medicine.

Another approach colloquially called ‘cell sheets’ has made large strides in the past two decades (Sakaguchi et al. 2015). Teruo Okano has mainly led this effort from Japan and they have shown that stackable cell sheets can have microvasculature if placed on top of a perfusable section of femoral tissue, or taking advantage of microchannels in a collagen gel (Asakawa et al. 2010; Sekine et al. 2013; Sakaguchi et al. 2013). These cell sheets can be stacked on top of each other in vitro making relatively thick constructs of around 100 μm. In vivo cell sheets can be stacked on top of each other following multiple surgeries to produce thicker pseudo tissue, although this is highly stressful to the animal (Shimizu et al. 2006). Stackable cell sheets have been very successful and have progressed through animal models of myocardial infarction, showing improvements in ejection fraction, even progressing onto pigs (Kawamura et al. 2012; Kawamura et al. 2013; Kawamura et al. 2017). Moreover, this approach has been approved through Japan’s fast-track system and is currently ready for a first in man trial.

## Considerations of using extra cell types within hydrogels

A few labs are now on the cusp of going into first in man trials with engineered constructs, with most labs being in agreement that cell types other than cardiomyocytes are beneficial. However, there are important observations that need to be considered to avoid the failures seen in previous clinical trials to this date. Special importance must be given to tracking non-cardiomyocyte cell numbers because these cells have a tendency to over-proliferate within the hydrogel. This effect can change ratios of cells and also make constructs that appear like fibrous tissue in histology. Also another important aspect to consider is that even the best hPSC-CM differentiations contain 3–5% non-cardiomyocytes of unspecified phenotype, which may explain different results between groups. Moreover, the paracrine vs contractility hypotheses of how these constructs produce beneficial effects in infarction models needs to be investigated thoroughly. If these constructs help by giving mechanical and contractile support, then their force generation and/or stiffness needs to be improved. If the paracrine hypothesis is favoured then the mechanism of this effect needs further investigation.

## Further candidate cell types for tissue engineering

Two cell types whose properties have not yet been taken advantage of in tissue engineering cardiac constructs are epicardium and endocardial cells. The epicardium is located around the outside of the heart and is a dynamic cell layer that contributes to non-myocyte lineages like: fibroblasts, vascular smooth muscle cells, and endothelial cells which have important roles in the coronary endothelium (Dettman et al. 1998; Gittenberger-de Groot 1998; Manner 1999; Mikawa and Gourdie 1996; Perez-Pomares et al. 2002; Zhou et al. 2008). Moreover, this cell layer has important paracrine functions and also contributes different cell types to the heart. The epicardium contributes to these multiple cell types by epithelial to mesenchymal transition during development and also during heart regeneration after insult in lower organisms (reviewed in Cao and Poss 2018). Therefore, its importance in cardiac development and also regeneration of heart tissue after injury cannot be overestimated, and taking advantage of this cell type could benefit tissue engineering of heart constructs greatly.

## Epicardial cells in tissue engineering

Studies that have investigated the effect of epicardial cells on cardiomyocytes have mostly been in vivo where the epithelium has an effect during development and regeneration. In vitro there are only a few studies which have focussed on epicardial cells, and this field has not progressed into tissue engineering avenues. In vitro epicardial cells have been isolated and hold many of the same characteristics as cells in vivo (van Tuyn et al. 2007; Bax et al. 2011; Moerkamp et al. 2016). These cells can undergo epithelial to mesenchymal transition upon stimulation with TGF-β and adult epicardial cells can form relatively large tubes. The interaction of isolated epicardial cells and cardiomyocytes has been investigated with early work suggesting that epicardial cells have an effect on proliferation of immature cardiomyocytes (Chen et al. 2002). It has also been shown that endothelial to mesenchymal transition of human epicardial cells bridging two populations of rat neonatal cardiomyocytes has a large effect on conduction, with vimentin positive epicardial cells slowing conduction considerably (Bax et al. 2011). Direct contact of epicardial cells isolated from quail with mouse neonatal cardiomyocytes showed an increase in cardiomyocyte proliferation, protein expression, and alignment but this was not due to effectors secreted into the media (Weeke-Klimp et al. 2010). Epicardial co-culture in a hydrogel with fibroblasts and HL-1 cells showed that the pro-inflammatory cytokine profile may be reduced and this could have enormous benefits for regenerative medicine approaches in the future (Becker et al. 2018).

Multiple papers have shown the epicardium is extremely important in the developing and regenerating heart. Having a pool of PSC derived epicardium cells would be of great interest for tissue engineering because they would be a potentially unlimited resource. Differentiation of epicardium cells from PSCs has recently been achieved by inducing cardiac progenitors and then differentiating into epicardial cells (Witty et al. 2014; Iyer et al. 2015). Now these cells can be generated under serum free conditions generating an almost pure population of cells which can undergo multiple population doublings (Bao et al. 2016; Zhao et al. 2017). Moreover, these cells have been shown to be able to be differentiated into cells resembling endocardium by applying VEGF, currently this protocol does not produce a high purity of CD31^+^ cells but these cells could be purified (Bao et al. 2017). Epicardial cells differentiated from PSCs and combined with differentiated cardiomyocytes in tissue engineered constructs were also shown to be effective at increasing fractional shortening after infarction in rats, and this combination of cell types was more effective than either cardiomyocytes or epicardial cells alone (Bargehr et al. 2017). These results show that epicardial cells have a positive effect on cardiomyocytes in vitro and that they could contribute significantly to tissue engineered constructs.

## Endocardial cells: an untapped resource in tissue engineering

Another cell type not being fully taken advantage of by tissue engineers is endocardial cells. The endocardium lines the inside region of the heart and forms from early cardiac mesoderm. Early in development the heart is a linear tube with only the endocardium and the myocardium which is separated by cardiac jelly. Upon looping the heart develops four chambers and the endocardium forms the trabeculae. Reciprocal signalling to and from the endocardium and the myocardium are extremely important during early development and NRG-1/ErbB2/B4, VEGF, Notch, FGF, and Ang-1 have all been implicated in myocardium proliferation and maturation (Tian and Morrisey 2012). Little work has been done to understand the potential role of endocardial cells is cardiac tissue engineered constructs.

## Tissue engineering for making chambers resembling ventricles

The perennial goal of tissue engineering is to make heart tissue as similar to in vivo myocardium as possible. Strides forward have been made recently by generation of 3D constructs which have been compared to 2D monolayer techniques with a comprehensive study showing that the 3D environment has a positive effect on maturation of cardiomyocytes (Tiburcy et al. 2017). Moreover, 3D engineered tissues combined with mechanical, electrical, or dynamic stimulation can further increase the maturity of these constructs (Tulloch et al. 2011; Hirt et al. 2014; Jackman et al. 2016; Ronaldson-Bouchard et al. 2018). Within these publications almost adult like contraction force, conduction velocity, and structural elements including T-tubules have been demonstrated.

The heart being a complex multicellular organ cannot be fully replicated in the lab with basic engineered constructs but needs four chambers, with valves and perfusion to the ventricles and to the myocardium itself. There have been a few attempts at making constructs more like hearts by tissue engineering methods. Ott et al. showed that the rat heart can be de-cellularised and then reseeded with cells to produce measurable parameters, this work has also been up scaled to the human heart (Ott et al. 2008; Guyette et al. 2016). However, these works suffer from using only cardiomyocytes to reanimate the heart and no other cell types, moreover, this approach relies upon having a heart to de-cellularise. Hydrogels have also been made with a chamber cultured with smooth muscle cells and neonatal cardiomyocytes around a silicone bulb, and this idea was further carried forward to produce a small chamber seeded with hPSC-CM (Gonen-Wadmany et al. 2004; Li et al. 2018). Ejection fractions are still not near adult human levels but this points to an interesting development in the field especially now since iPSC-CM can be produced in large amounts relatively easily. A small chamber with a valve was also made by using electrospun PCL/gelatin nanofibres into the shape of a ventricle seeded with neonatal rat cardiomyocytes or hPSC-CM (MacQueen et al. 2018). These approaches show that the beginning of making whole hearts can be achieved but the goal has to be on making engineered constructs as close to the myocardium as possible, and therefore a focus on producing a multicellular perfusable piece of heart tissue is paramount.

## Conclusion

The heart is extremely complex and the myocardium not only has multiple cell types with specific functions but it is primarily a mechanical pump. These two factors combined present the cell biologist and the engineer a truly fascinating problem which will need multi-disciplinary approach, if anything similar to an actual heart will be made in the lab. Here we have outlined the current approaches being used to answer this question and have highlighted that a true multi-cellular preparation similar to the heart with vasculature and cardiomyocytes does not yet exist. Currently, cell biologists are becoming better at making high volumes of cells which can be directed towards specific lineages, and this could make the approach viable in the future. If successful, this could have multiple applications including the field of regenerative medicine, drug discovery, or disease modelling.

## References

[CR1] Abramochkin DV, Lozinsky IT, Kamkin A (2014). Influence of mechanical stress on fibroblast-myocyte interactions in mammalian heart. J Mol Cell Cardiol.

[CR2] Asakawa N, Shimizu T, Tsuda Y, Sekiya S, Sasagawa T, Yamato M, Fukai F, Okano T (2010). Pre-vascularization of in vitro three-dimensional tissues created by cell sheet engineering. Biomaterials.

[CR4] Bao X, Lian X, Hacker TA, Schmuck EG, Qian T, Bhute VJ, Han T, Shi M, Drowley L, Plowright A, Wang QD, Goumans MJ, Palecek SP (2016). Long-term self-renewing human epicardial cells generated from pluripotent stem cells underdefined xeno-free conditions. Nat Biomed Eng.

[CR5] Bao X, Bhute VJ, Han T, Qian T, Lian X, Palecek SP (2017). Human pluripotent stem cell-derived epicardial progenitors can differentiate to endocardial-like endothelial cells. Bioeng Transl Med.

[CR6] Bargehr J, Hofsteen P, Bhandari S, Gambardella L, Ong L, Iyer D, Sampaziotis F, Weinberger F, Martinson M, Bernard W, Figg N, Bennett M, Murry CE, Sinha S (2017). Human embryonic stem cell derived epicardial cells advance cardiomyocyte-based heart regeneration. Eur Heart J.

[CR7] Basnett P, Marcello E, Lukasiewicz B, Panchal B, Nigmatullin R, Knowles JC, Roy I (2018). Biosynthesis and characterization of a novel, biocompatible medium chain length polyhydroxyalkanoate by Pseudomonas mendocina CH50 using coconut oil as the carbon source. J Mater Sci Mater Med.

[CR8] Bax NA, Pijnappels DA, van Oorschot AA, Winter EM, de Vries AA, van Tuyn J, Braun J, Maas S, Schalij MJ, Atsma DE, Goumans MJ, Gittenberger-de Groot AC (2011). Epithelial-to-mesenchymal transformation alters electrical conductivity of human epicardial cells. J Cell Mol Med.

[CR9] Becker M, Maring JA, Schneider M, Herrera Martin AX, Seifert M, Klein O, Braun T, Falk V, Stamm C (2018). Towards a novel patch material for cardiac applications: tissue specific extracellular matrix introduces essential key features to decellularized amniotic membrane. Int J Mol Sci.

[CR10] Bilic J, Izpsua Belmonte JC (2012). Concise review: induced pluripotent stem cells versus embryonic stem cells: close enough or yet too far apart?. Stem Cells.

[CR11] Cao J, Poss KD (2018). The epicardium as a hub for heart regeneration. Nat Rev Cardiol.

[CR12] Carrier L, Paradaki M, Rupnick M, Schoen FJ, Bursac N, Langer R, Freed LE, Vunjak-Novakovich G (1999). Cardiac tissue engineering cell seeding cultivation parameters and tissue contruct characterization. Biotechnol Bioeng.

[CR13] Cartledge JE, Kane C, Dias P, Tesfom M, Clarke L, Mckee B, AlAyoubi S, Chester A, Yacoub MH, Camelliti P, Terracciano CM (2015). Functional crosstalk between cardiac fibroblasts and adult cardiomyocytes by soluble mediators. Cardiovasc Res.

[CR14] Chen TH, Chang TC, Kang JO, Choudhary B, Makita T, Tran CM, Burch JB, Eid H, Sucov HM (2002). Epicardial induction of fetal cardiomyocyte proliferation via a retinoic acid-inducible trophic factor. Dev Biol.

[CR15] Chiu LL, Montgomery M, Liang Y, Liu H, Radisic M (2012). Perfusable branching microvessel bed for vascularization ofengineered tissues. Proc Natl Acad Sci USA.

[CR16] Denning C, Borgdorff V, Crutchley J, Firth KS, Goerge V, Kalra S, Kpmdrashov A, Hoang MD, Mosqueira D, Patel A, Prodanov L, Rajamohan D, Skarmes WC, Smith JG (1863). Young LE (2016) Cardiomyocytes from human pluripotent stem cells: from laboratory curiosity to industrial biomedical platform. Biochim Biophys Acta.

[CR17] Dettman RW, Denetclaw W, Ordahl CP, Bristow J (1998). Common epicardial origin of coronary vascular smooth muscle, perivascular fibroblasts, and intermyocardial fibroblasts in the avian heart. Dev Biol..

[CR18] Eschenhagen T, Zimmermann WH (2005). Engineering myocardial tissue. Circ Res.

[CR19] Gittenberger-de Groot AC, Vrancken Peeters MP, Mentink MM, Gourdie RG, Poelmann RE (1998). Epicardium-derived cells contribute a novel population to the myocardial wall and the atrioventricular cushions. Circ Res.

[CR20] Gonen-Wadmany M, Gepstein L, Seliktar D (2004). Controlling the cellular organization of tissue-engineered cardiac constructs. Ann N Y Acad Sci.

[CR21] Guyette JP, Charest JM, Mills RW, Jank BJ, Moser PT, Gilpin SE, Gershlak JR, Okamoto T, Gonzalez G, Milan DJ, Gaudette GR, Ott HC (2016). Bioengineering human myocardium on native extracellular matrix. Circ Res.

[CR22] Hirt MN, Boeddinhaus J, Mitchell A, Schaaf S, Bornchen C, Muller C, Schulz H, Hubner N, Stenzig J, Stoehr A, Neuber C, Eder A, Luther PK, Hansen A, Eschenhagen T (2014). Functional improvement and maturation of rat and human engineered heart tissue by chronic electrical stimulation. JMCC.

[CR24] Iyer D, Gambardella L, Bernard WG, Serrano F, Mascetti VL, Pedersen RA, Talasila A, Sinha S (2015). Robust derivation of epicardium and its differentiated smooth muscle cell progeny from human pluripotent stem cells. Development.

[CR73] Jackman CP, Carlson AL, Bursac N (2016). Dynamic culture yields engineered myocardium with near-adult functional output. Biomaterials.

[CR25] Jugdutt BI (2003). Remodeling of the myocardium and potential targets in the collagen degradation and synthesis pathways. Curr Drug Targets Cardiovasc Haematol Disord.

[CR26] Kawamura M, Miyagawa S, Miki K, Saito A, Fukushima S, Higuchi T, Kawamura T, Kuratani T, Daimon T, Shimizu T, Okano T, Sawa Y (2012). Feasibility, safety, and therapeutic efficacy of human induced pluripotent stem cell-derivedcardiomyocyte sheets in a porcine ischemic cardiomyopathy model. Circulation.

[CR27] Kawamura M, Miyagawa S, Fukushima S, Saito A, Miki K, Ito E, Sougawa N, Kawamura T, Daimon T, Shimizu T, Okano T, Toda K, Sawa Y (2013). Enhanced survival of transplanted human induced pluripotent stem cell-derived cardiomyocytes by the combination of cell sheets with the pedicled omental flap technique in a porcine heart. Circulation.

[CR28] Kawamura M, Miyagawa S, Fukushima S, Saito A, Miki K, Funakoshi S, Yoshida Y, Yamanaka S, Shimizu T, Okano T, Daimon T, Toda K, Sawa Y (2017). Enhanced therapeutic effects of human iPS cell derived-cardiomyocyte by combined cell-sheets with omental flap technique in porcine ischemic cardiomyopathy model. Sci Rep.

[CR29] Kenash G, Roa Lara A, Dahlmann J, Zweigerdt R, Sscwanke K, Hegermann J, Skvorc D, Gawol A, Azizian A, Wagner S, Maier LS, Krause A, Drager G, Ochs M, Haverich A, Gruh I, Martin U (2013). Murine and human pluripotent stem cell-derived cardiac bodies form contractile myocardial tissue in vitro. Eur Heart J.

[CR32] Krenning G, Zeisberg EM, Kalluri R (2010). The origin of fibroblasts and mechanism of cardiac fibrosis. J Cell Physiol.

[CR33] Leor J, Aboulafai-Etzion S, Dar A, Shapiro L, Barbash IM, Battler A, Granot Y, Cohen S (2000). Bioengineered cardiac graphs: a new approach to repair the infarcted myocardium. Circulation.

[CR34] Lesman A, Habib M, Caspi O, Gepstein A, Aarbel G, Levenberg S, Gepstein L (2010). Transplantation of a tissue engineered human vascularized cardiac muscle. Tissue Eng Part A.

[CR35] Li RK, Jia ZQ, Weisel RD, Mickle DA, Choi A, Yau TM (1999). Survival and function of bioengineered cardiac grafts. Circulation.

[CR36] Li Y, Asfour H, Bursac N (2017). Age-dependent functional crosstalk between cardiac fibroblasts and cardiomyocytes in a 3D engineered cardiac tissue. Acta Biomater.

[CR37] Li RA, Keung W, Cashman TJ, Backeris PC, Johnson BV, Bardot ES, Wong AOT, Chan PKW, Chan CWY, Costa KD (2018). Bioengineering an electro-mechanically functional miniature ventricular heart chamber from human pluripotent stem cells. Biomaterials.

[CR39] Liau B, Christoforou N, Leong KW, Bursac N (2011). Pluripotent stem cell-derived cardiac tissue patch with advanced structure and function. Biomaterials.

[CR40] Liau B, Jackman CP, Li Y, Bursac N (2017). Developmental stage-dependent effects of cardiac fibroblasts on function of stem cell-derived engineered cardiac tissues. Sci Rep.

[CR41] MacQueen LA, Sheehy SP, Chantre CO, Zimeerman JF, Pasqualini FS, Liu X, Goss JA, Campbell PH, Gonzalez GM, Park SJ, Capulli AK, Ferrier JP, Kosar TF, Mahadevan L, Pu WT, Parker KK (2018). A tissue-engineered scale model of the heart ventricle. Nat Biomed Eng.

[CR42] Manner J (1999). Does the subepicardial mesenchyme contribute myocardioblasts to the myocardium of the chick embryo heart? A quail-chick chimera study tracing the fate of the epicardial primordium. Anat Rec.

[CR43] Marsano A, Maidhof R, Wan LQ, Wang Y, Gao J, Tandon N, Vunjak-Novakovic G (2010). Scaffold stiffness affects the contractile function of three-dimensional engineered cardiac constructs. Biotechnol Prog.

[CR44] Mikawa T, Gourdie RG (1996). Pericardial mesoderm generates a population of coronary smooth muscle cells migrating into the heart along with ingrowth of the epicardial organ. Dev Biol.

[CR45] Moerkamp AT, Lodder K, van Herwaarden T, Dronkers E, Dingenouts CK, Tengström FC, van Brakel TJ, Goumans MJ, Smits AM (2016). Human fetal and adult epicardial-derived cells: a novel model to study their activation. Stem Cell Res Ther.

[CR47] Morritt AN, Bortolotto SK, Dilley RJ, Han X, Kompa AR, McCombe D, Wright CE, Itescu S, Angus JA, Morrison WA (2007). Cardiac tissue engineering in an in vivo vascularized chamber. Circulation.

[CR48] Ott HC, Matthiesen TS, Goh SK, Black LD, Kren SM, Netoff TI, Taylor DA (2008). Perfusion-decellularized matrix: using nature’s platform to engineer a bioartificial heart. Nat Med.

[CR49] Perez-Pomares JM, Carmona R, Gonzalez-Iriarte M, Atencia G, Wessels A, Munoz-Chapuli R (2002). Origin of coronary endothelial cells from epicardial mesothelium in avian embryos. Int J Dev Biol.

[CR50] Pinto AR, Ilinykh A, Ivey MJ, Kuwabara JT, D’antoni ML, Debuque R, Chandran A, Wang L, Arora K, Rosenthal NA, Tallquist MD (2016). Revisiting cardiac cellular composition. Circ Res.

[CR51] Porter KE, Turner NA (2009). Cardiac fibroblasts: at the heart of myocardial remodeling. Pharmacol Ther.

[CR52] Radisic M, Park H, Shing H, Consi T, Schoen FJ, Langer R, Freed LE, Vunjak-Novakovic G (2004). Functional assembly of engineered myocardium by electrical stimulation of cardiac myocytes cultured on scaffolds. Proc Natl Acad Sci USA.

[CR53] Rohr S (2011). Cardiac fibroblasts in cell culture systems: myofibroblasts all along?. J Cardiovasc Pharmacol.

[CR54] Ronaldson-Bouchard K, Ma SP, Yeager K, Chen T, Song L, Sirabella D, Morikawa K, Teles D, Yazawa M, Vunjak-Novakovic G (2018). Advanced maturation of human cardiac tissue grown from pluripotent stem cells. Nature.

[CR55] Sakaguchi K, Shimizu T, Horaguchi S, Sekine H, Yamato M, Umezu M, Okano T (2013). In vitro engineering of vascularized tissue surrogates. Sci Rep.

[CR56] Sakaguchi K, Shimizu T, Okano T (2015). Construction of three-dimensional vascularized cardiac tissue with cell sheet engineering. J Control Release.

[CR57] Sekine H, Shimzu T, Sakaguchi K, Dobashi I, Wada M, Yamato M, Kobayashi E, Umezu M, Okano T (2013). In vitro fabrication of functional three-dimensional tissues with perfusable blood vessels. Nat Commun.

[CR58] Shimizu T, Sekine H, Yang J, Isoi Y, Yamato M, Kikuchi A, Kobayashi E, Okano T (2006). Polysurgery of cell sheet grafts overcomes diffusion limits to produce thick vascularized myocardial tissues. FASEB.

[CR59] Shinde AV, Frangogiannis NG (2014). Fibroblasts in myocardial infarction: a role in inflammation and repair. J Mol Cell Cardiol.

[CR60] Stoehr A, Hirt MN, Hansen A, Seiffert M, Conradi L, Uebeler J, Limbourg FP, Eschenhagen T (2016). Spontaneous formation of extensive vessel-like structures in murine engineered heart tissue. Tissue Eng Part A.

[CR61] Sun X, Altalhi W, Nunes SS (2016). Vascularization strategies of engineered tissues and their application in cardiac regeneration. Adv Drug Deliv Rev.

[CR62] Sweeney M, Foldes G (2018). It takes two: endothelial-perivascular cell cross-talk in vascular development and disease. Front Cardiovasc Med..

[CR63] Tang J, Wang J, Huang K, Ye Y, Su T, Qiao L, Hensley MT, Caranasos TG, Zhang J, Gu Z, Cheng K (2018). Cardiac cell-integrated microneedle patch for treating myocardial infarction. Sci Adv.

[CR64] Tian T, Morrisey EE (2012). Importance of myocyte-nonmyocyte interactions in cardiac development and disease. Circ Res.

[CR65] Tiburcy M, Hudson JE, Balfanz P, Schlick S, Meyer T, Chang Liao ML, Levent E, Raad F, Zeidler S, Wingender E, Riegler J, Wang M, Gold JD, Kehat I, Wettwer E, Ravens U, Dierickx P, Van Laake LW, Goumans MJ, Khadjeh S, Toischer K, Hasenfuss G, Couture LA, Unger A, Linke WA, Araki T, Neel B, Keller G, Gepstein L, Wu JC, Zimmermann WH (2017). Defined engineered human myocardium with advanced maturation for applications in heart failure modeling and repair. Circulation.

[CR66] Tulloch NL, Muskheli V, Razumova MV, Korte FS, Regnier M, Hauch KD, Pabon L, Reinecke H, Murry CE (2011). Growth of engineered human myocardium with mechanical loading and vascular coculture. Circ Res.

[CR67] van Tuyn J, Atsma DE, Winter EM, van der Velde-van Dijke I, Pijnappels DA, Bax NA, Knaän-Shanzer S, Gittenberger-de Groot AC, Poelmann RE, van der Laarse A, van der Wall EE, Schalij MJ, de Vries AA (2007). Epicardial cells of human adults can undergo an epithelial-to-mesenchymal transition and obtain characteristics of smooth muscle cells in vitro. Stem Cells..

[CR68] Weeke-Klimp A, Bax NA, Bellu AR, Winter EM, Vrolijk J, Plantinga J, Maas S, Brinker M, Mahtab EA, Gittenberger-de Groot AC, van Luyn MJ, Harmsen MC, Lie-Venema H (2010). Epicardium-derived cells enhance proliferation, cellular maturation and alignment of cardiomyocytes. J Mol Cell Cardiol.

[CR69] Weinburger F, Breckwoldt K, Pecha S, Kelley A, Geertz B, Starbatty J, Yorgen T, Cheng KH, Lessmann K, Stolen T, Scherrer-Crosbie M, Smith G, Reichenspurner H, Hansen A, Eschenhagen T (2016). Cardiac repair in guinea pigs with human engineered heart tissue from induced pluripotent stem cells. Sci Transl Med.

[CR70] Witty AD, Mihic A, Tam RY, Fisher SA, Mikryukov A, Shoichet MS, Li RK, Kattman SJ, Keller G (2014). Generation of the epicardial lineage from human pluripotent stem cells. Nat Biotechnol.

[CR71] Zhao J, Cao H, Tian L, Huo W, Zhai K, Wang P, Ji G, Ma Y (2017). Efficient differentiation of TBX18^+^/WT1^+^ epicardial-like cells from human pluripotent stem cells using small molecular compounds. Stem Cells Dev.

[CR72] Zhou B, Ma Q, Rajagopal S, Wu SM, Domian I, Rivera-Feliciano J, Jiang D, von Gise A, Ikeda S, Chien KR, Pu WT (2008). Epicardial progenitors contribute to the cardiomyocyte lineage in the developing heart. Nature.

